# Practical Essays on Medical Education, and the Medical Profession in the United States

**Published:** 1831

**Authors:** 


Art. VIII.—*Practical Essays on Medical Education, and the Medical Profession, in the United States.* By Daniel Drake, M. D., Professor in the Medical College of Ohio.—Cincinnati, Roff & Young.

The Essays originally published by Dr. Drake in this Journal, on the subject of Medical Education, and the Medical Profession, have been republished by the author in a separate form, with three additional Essays—On the Causes of Error in the Medical and Physical Sciences—On Legislative Enactments—On Professional Quarrels. Any recommendation of this work from us, would be superfluous. The Essay on the Causes of Error in the Med. and Phys. Sciences has been written by the author in one of his happiest moods, and should be read with care by every one interested in the advancement of the honor and dignity of the profession. F.

